# Dr. Cammann’s Stethoscope

**Published:** 1855-04

**Authors:** 


*Dr. Cammann's Stethoscope.*—This new instrument consists of an objective end of ebony, two inches in diameter at its base, narrowing above, till it branches into two tubes of gum elastic and metallic wire, for the purpose of flexibility. These are connected with two curved tubes of German silver, terminating in ivory knobs. The two branching tubes are connected by an elastic spring.

The object of the arrangement is this: The ivory knobs being fitted into the ears of the auscultator, the trumpet-shaped objective end is applied to the patient, the sounds being conducted through an even bore of two and a half lines in diameter. Its advantages are the use of both ears, the isolation of the sound from all other noises, the increase in the intensity of the sound, and the leaving both hands of the examiner free.

The New York Medical Times says that "One point, hitherto *sub judice*, is settled by this instrument, viz., that the sound is conducted entirely through the air, and not at all through the media, as these were, for experiment's sake, changed nine or ten times, without affecting in the least the intensity of the conducted sound. On making the objective end solid, all sound was lost."

If carefully constructed, we must look upon this instrument as a very valuable addition to diagnostic art. H.

---

Dr. John McCall, in his remarks on the diseases of Oneida county, read before the society of that county, asserts that he has not bled a patient from the arm during fourteen years. Such a remark shows the tendency of the medical mind at present, and the changes wrought in it of late years. At the same time it was hardly a prudent speech to make. Dr. McCall may have found it necessary to bleed within a day after reading his statement, and doubtless, if it were necessary, would do so unhesitatingly; but it may be that some tyro listening to him, has been induced thereby to let slip the golden opportunity of saving a life. H.

